# Highly Sensitive UV Photodiode Composed of β-Polyfluorene/YZnO Nanorod Organic-Inorganic Hybrid Heterostructure

**DOI:** 10.3390/nano10081486

**Published:** 2020-07-29

**Authors:** Youngmin Lee, Soo Youn Kim, Deuk Young Kim, Sejoon Lee

**Affiliations:** 1 Quantum-Functional Semiconductor Research Center, Dongguk University-Seoul, Seoul 04623, Korea; ymlee@dongguk.edu (Y.L.); dykim@dongguk.edu (D.Y.K.); 2 Division of Physics & Semiconductor Science, Dongguk University-Seoul, Seoul 04623, Korea; sooyoun@dgu.ac.kr

**Keywords:** Y-doped zinc oxide, nanorod, polyfluorene, hybrid structure, heterojunction, photodiode

## Abstract

The highly sensitive ultra-violet (UV) photodiode was demonstrated on the organic-inorganic hybrid heterostructure of β-phase p-type polyfluorene (PFO)/n-type yttrium-doped zinc oxide nanorods (YZO-NRs). The device was fabricated through a simple fabrication technique of β-phase PFO coating onto YZO-NRs that had been directly grown on graphene by the hydrothermal synthesis method. Under UV illumination (λ = 365 nm), the device clearly showed excellent photoresponse characteristics (e.g., high quantum efficiency ~690%, high photodetectivity ~3.34 × 10^12^ cm·Hz^1/2^·W^−1^, and fast response time ~0.17 s). Furthermore, the ratio of the photo current-to-dark current exceeds 10^3^ even under UV illumination with a small optical power density of 0.6 mW/cm^2^. We attribute such superb photoresponse characteristics to both Y incorporation into YZO-NRs and conformation of β-phase PFO. Namely, Y dopants could effectively reduce surface states at YZO-NRs, and β-phase PFO might increase the photocarrier conductivity in PFO. The results suggest that the β-phase p-PFO/n-YZO-NR hybrid heterostructure holds promise for high-performance UV photodetectors.

## 1. Introduction

For the last two decades, ZnO nanostructures have garnered substantial attention because of their prodigious potential for blue and ultra-violet (UV) optoelectronic devices [1,2,3,4,5,6]. Among various nanostructures, 1-dimensional ZnO (e.g., nanorod [7,8], nanoneedle [9,10], nanopillar [11,12], etc.) is one of the most attractive nanoarchitectures due to its short pathway for carrier transport [13], high surface-to-volume ratio for photon collection [14], and low exciton-phonon coupling strength [15]. In recent years, furthermore, extra foreign element-doped ZnO nanorods (NRs) have been of particular interest because extra foreign dopants could improve the electrical and the optical properties of ZnO [16,17,18,19,20,21,22,23,24]. For instance, we recently reported that Y-doping led to the reduction of oxygen-related defects in YZnO (YZO) [22,23]; hence, the electrical conductivity and the excitonic emission properties could be improved by Y doping into YZO films [19,20] and n-type yttrium-doped zinc oxide nanorods (YZO-NRs) [21,22,23], respectively.

In addition, ZnO is highly compatible with other materials and it can be categorized into two different aspects. One is an availability for the easy growth of single-crystalline ZnO; i.e., high-quality ZnO can be effectively grown on every substrate such as Si [25,26], GaN [27,28], glass [29], plastic substrates [30,31], and graphene [32,33,34]. Among various possible substrates, particularly, 2-dimensional graphene offers a special benefit because high-quality ZnO could be directly grown on graphene due to its honeycomb lattices, which act as the seed sites for nucleation of wurtzite ZnO [33,34,35]. The other is that ZnO could be hybridized with various organic semiconductors. This allows us to form novel functional device architectures that can open up new avenues toward various applications using ZnO-based inorganic-organic hybrid heterostructures. For instance, multi-level memory cells [36,37], high speed barristors [38], color-tunable light-emitting diodes [18,39], and color-dependent photodiodes [40] are typical examples of ZnO-based inorganic-organic hybrid electronic and optoelectronic devices. Among various organic semiconductors, β-phase polyfluorene (PFO) is of ample interest particularly for optoelectronic device applications because of its wide band-gap energy and good processability [41,42,43]. Furthermore, since β-phase PFO has a higher charge-carrier mobility than other organic semiconductors [44], many researchers have been devoted to demonstrating highly efficient solid-state light-emitting devices using the inorganic-organic hybrid heterostructure of ZnO/PFO [45,46,47,48,49]. To our best awareness, however, ZnO/PFO-based light-sensing photodiodes (PDs) have rarely been investigated [50,51,52] in spite of great potential for highly sensitive UV detection (e.g., space exploration, environmental monitoring, flame detection, defense warning, medical equipment, security communication systems, etc. [1,2,3,4]).

Aiming at demonstrating the high-performance UV PDs by using PFO and YZO-NRs, we have therefore investigated the fabrication and the characterization of the β-phase p-type PFO/n-type YZO-NRs organic-inorganic hybrid heterojunction PDs. The hybrid PDs were effectively fabricated by spin-coating of β-phase PFO onto YZO-NRs that had been directly grown on graphene by the hydrothermal synthesis method. The devices exhibited excellent UV photoresponse properties even under low-power UV illumination. Herein, we report on experimental data obtained from systematic analyses of the material-to-device characteristics.

## 2. Experimental Section

### 2.1. Preparation of Graphene/SiO_2_/Si Substrate

**Figure 1:**
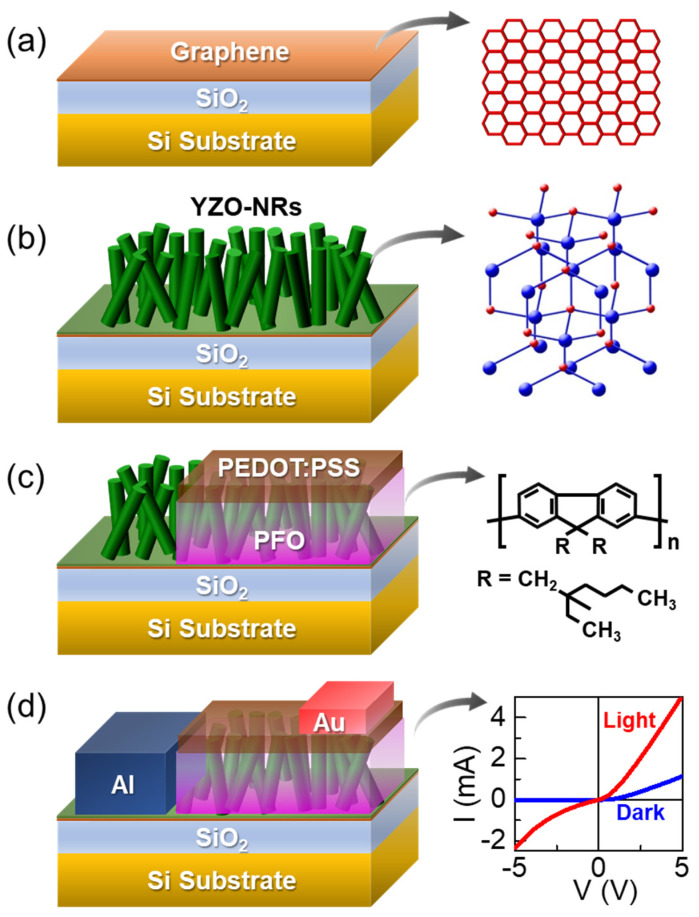
Experimental procedures for the fabrication of the PFO/YZO-NR PD: (**a**) graphene transfer onto the SiO_2_/Si substrate, (**b**) growth of YZO-NRs on graphene by the hydrothermal method. (**c**) spin-coating of PFO and PEDOT:PSS on YZO-NRs, and (**d**) Formation of Ohmic electrodes via Al and Au evaporation. The right-hand-side insets of (**a**–**d**) represent the honeycomb lattices of graphene, the wurtzite lattices of YZO, the molecule chain structure of PFO, and I–V characteristic curve of the PFO/YZO-NR PD, respectively. YZO-NRs: n-type yttrium-doped zinc oxide nanorods; PD: photodiodes; PFO: polyfluorene.

Figure 1 displays the schematic illustrations of the device fabrication procedure. As an initial task, we prepared the graphene/SiO_2_/Si substrate for the direct growth of high-quality YZO-NRs. First, the single-layer graphene sheet was grown on Cu foil by chemical vapor deposition [53], and transferred onto the SiO_2_/Si substrate through the typical transfer method using poly(methyl methacrylate) (Figure 1a). Then, the substrate was subsequently annealed at 280 °C for 10 min in vacuum to eliminate residual chemical contaminants on the graphene surface [32].

### 2.2. Direct Growth of YZO-NRs on Graphene

The hexagonal YZO-NRs were directly grown on graphene via the seedless hydrothermal method (Figure 1b). First, the aqueous solution of YZO (0.1 M) was prepared by mixing zinc nitrate hexahydrate (Zn(NO_3_)_2_∙6H_2_O), hexamethylenetetramine (C_6_H_12_N_4_), and yttrium acetate hydrate (Y(CH_3_COO)_3_∙H_2_O) in deionized (DI) water (35 mL). Here, we note that the molar fraction ratio of Zn:Y was varied from 99%:1% to 95%:5% to investigate the effects of Y contents on both the material and the device characteristics. After transferring the prepared solution into the autoclave, the YZO-NRs were hydrothermally synthesized at 85 °C for 8 h. Thereafter, the samples were rinsed in DI water to clean the precipitates off the surface, and cured at 300 °C for 10 min in an electric oven. Finally, to improve the crystal quality of YZO, the samples were annealed at 500 °C for 1 min in vacuum by rapid thermal annealing.

### 2.3. Fabrication of PFO/YZO-NR PDs

The p-PFO/n-YZO-NR organic-inorganic hybrid heterostructures were fabricated by spin-coating of PFO (poly(9,9′-dioctyfluorene) onto YZO-NRs (Figure 1c). First, PFO (50 mg) was dissolved in chloroform (1 mL). The mixture solution was coated on the YZO-NR samples at a spinning speed of 1000 rpm; then, the samples were heated at 120 °C for 10 min in an electrical oven to transform the casted PFO layer into β-phase. In order to enhance the hole carrier injection probability in the hybrid structure, additionally, we spin-coated the hole injection layer of PEDOT:PSS on the top of PFO at 4000 rpm. After dehydrating the samples at 110 °C for 40 min, finally, the Ohmic electrodes of Al and Au were patterned on YZO and PEDOT:PSS, respectively, by thermal evaporation (Figure 1d). We here note that the active area for photon collection was designed to be 1 cm^2^.

### 2.4. Characterization of Materials and Devices

The topographic properties of YZO-NRs and PFO were monitored by scanning electron microscopy (SEM) using an FE SEM XL-30 system (Phillips, Eindhoven, The Netherlands), and the optical properties of the samples were characterized by photoluminescence (PL) spectroscopy using a home-built PL system equipped with an excitation source of the He-Cd laser (λ = 325 nm) and a 75-cm monochromator (GaAs photomultiplier tube). The electrical properties of the PFO/YZO-NR PDs were examined by using a semiconductor device parameter analyzer of Keysight B1500A (Keysight Technologies, Santa Rosa, CA, USA). The UV photoresponse properties of the devices were assessed under UV illumination (λ = 365 nm) with the optical power density (P_opt_) of 0–0.6 mW/cm^2^.

## 3. Results and Discussion

**Figure 2:**
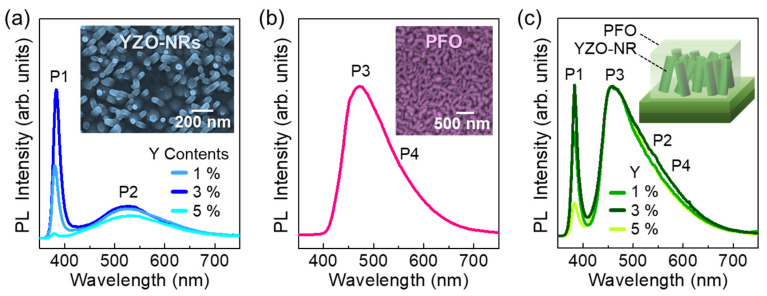
Optical properties of the prepared materials: (**a**) PL spectra of YZO-NRs with the different Y contents (1%–5%), (**b**) PL spectrum of PFO, and (**c**) PL spectra for the hybrid heterostructures of the PFO/YZO-NR (Y: 1%–5%). The inset of (**a**) and (**b**) display the SEM images of YZO-NRs and PFO, respectively; and the inset of (**c**) illustrates the PFO/YZO-NR heterostructure.

Since both graphene and zinc oxide have a hexagonal lattice configuration along the *a*-plane [54,55], the YZO-NRs were effectively grown on graphene even though no seed layer was used for the hydrothermal synthesis process (see the inset of Figure 2a). However, each nanorod has an irregular direction, presumably, because of the difference in *a*-axis lattice constants between zinc oxide (≅3.25 Å) and graphene (≅2.46 Å) [54,55]. Despite such an arbitrary direction of YZO-NRs, most of the nanorods possess a high aspect ratio (i.e., *l*_ave_ ~ 40 nm and *d*_ave_ ~ 250 nm) that may give rise to the increase in the photon collection area (i.e., large surface-to-volume ratio) for the PFO/YZO-NR PD.

Figure 2a shows the optical properties of the YZO-NRs (Y: 1–5%). Regardless of the Y contents, all samples exhibit two predominant PL features at P1 ~ 380 nm and P2 ~ 530 nm. UV emission (i.e., P1) originates from the near band-edge radiative optical transition, and green emission (i.e., P2) arises from the deep-level transition, associating with oxygen-related defects (e.g., oxygen vacancies (V_O_)) in host material ZnO [21,22,23]. Compared to the YZO-NRs with Y = 1%, the sample with Y = 3% displays much stronger UV emission whereas deep level emission is almost comparable. This infers that the crystal quality of YZO was dramatically improved via use of an appropriate concentration of Y because the Y dopants act as the V_O_ compensators [20,23]. However, UV emission became significantly weak when the Y concentration was increased up to 5%. We impute such a degradation to the lattice distortion in YZO because the incorporation of the abundant Y dopants may not only create the Y–Y and/or Y–O clusters [56] but also cause the residual stress in the YZO crystal due to the larger ionic radius of Y^3+^ (0.92 Å) than that of Zn^2+^ (0.74 Å) [20].

The morphological and the optical properties of PFO are shown in Figure 2b. To investigate the intrinsic properties of PFO, we prepared the reference sample of the pristine PFO layer by coating it onto the SiO_2_/Si substrate. The PFO layer displays a grain-aggregated topography (see the inset of Figure 2b), resulting from the volume shrinkage during the thermal treatment [41]. For the PL characteristics, the PFO layer clearly reveals a huge blue-emission peak (P3) at ~470 nm with a broad hump (P4) at ~540 nm (Figure 2b). These correspond to the typical PL feature of *β*-phase PFO [41,43] that comprises a flatter geometry of backbone-like PFO molecules. Namely, the PFO layer was well-dispersed along the surface direction. This will increase the active contact area of PFO to YZO-NRs; and it may allow an effective formation of the stable heterojunction between p-PFO and n-YZO. Furthermore, the higher carrier mobility of *β*-phase PFO than that of glassy PFO [44] may also improve the photoresponse characteristics of the PFO/YZO-NR PD device. Figure 2c shows the PL spectra of the PFO/YZO-NRs hybrid heterostructures. The samples reveal no additional peaks, except for intrinsic PL properties from both YZO-NRs (i.e., P1 and P2) and PFO (i.e., P3 and P4). Although the samples exhibit a similar aspect in their PL spectra; as discussed above, the intensity ratio of P1 (i.e., UV) to P3 (i.e., green) is maximized in the sample doped with an appropriate fraction of Y (i.e., 3%).

**Figure 3:**
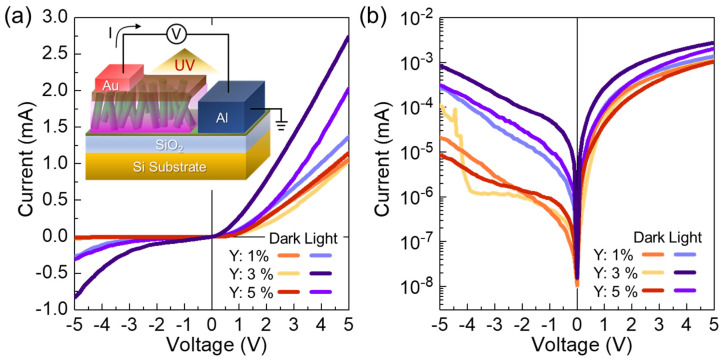
I–V characteristic curves of the PFO/YZO-NR PDs (Y: 15%) under dark and UV illumination (P_opt_ = 0.2 mW/cm^2^): (**a**) linear scale and (**b**) semi-logarithmic scale.

Next, we characterized the electrical characteristics of the PFO/YZO-NR PDs. To investigate the effect of the Y contents on the current-voltage (I–V) characteristics, we fabricated three different PFO/YZO-NR PD samples by using three different types of YZO-NRs (i.e., Y: 1%, 3%, and 5%). For convenience, we refer to those samples as Y1, Y3, and Y5, respectively. Under the dark state, as shown in Figure 3a, all of the samples clearly show the rectifying behavior with the turn-on voltage of ~0.75 V and the ideality factor of ~3.1. Such a large ideality factor might be attributed to the multiple current pathways, arising from the surface states created by native point defects on the YZO-NR surface [57]. When illuminating the 365-nm UV light (P_opt_ = 0.2 mW/cm^2^) onto the devices, the current level is considerably increased in both the positive and the negative bias regions. Particularly, the Y3 sample exhibits a higher photo-to-dark current ratio than Y1 and Y5 (Figure 3b). This signifies that an appropriate concentration of Y (i.e., 3%) can help increasing the photocarrier density, as discussed below.

**Figure 4:**
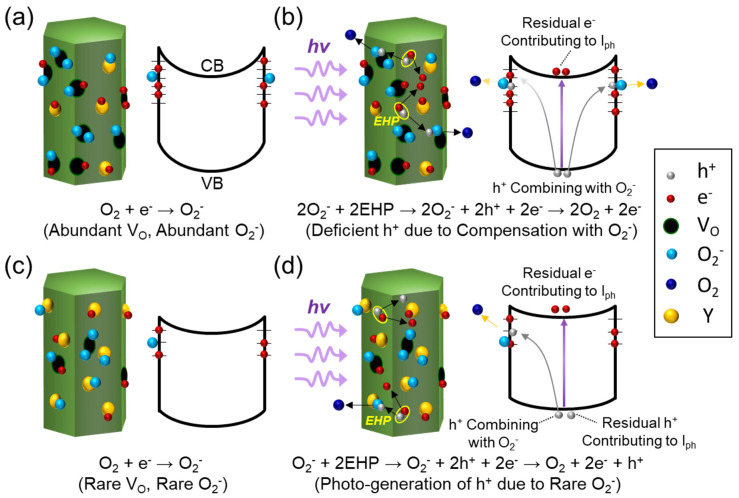
Schematic illustrations of the YZO-NR surface (left-hand-side) and its corresponding energy band diagram (right-hand-side): Defective YZO-NR with a lower Y concentration at (**a**) dark and (**b**) UV illumination, and less-defective YZO-NR with a moderate Y concentration at (**c**) dark and (**d**) UV illumination. The chemical formula in each figure denotes the chemical reaction under the given situation.

To explain such a hypothesis, we interpret the effect of the Y contents on the photocurrent of the PFO/YZO-NR PD. Figure 4 schematically represents the nanorod surfaces and their corresponding energy band diagrams for two different types of YZO-NRs; i.e., one contains a lower concentration of Y (Figure 4a,b), and the other has a moderate concentration of Y (Figure 4c,d). As aforementioned, the Y dopants play a key role as the V_O_ compensators [20,23]. Therefore, the former one might have a high concentration of negative charge carriers because a lower Y concentration would leave a large number of V_O_ at the nanorod surface. In this case, a lot of oxygen molecules would be adsorbed at the abundant V_O_ sites; hence, the large number of negatively charged oxygen ions could be created via bonding of O_2_ molecules with negative charge carriers at V_O_ sites (i.e., O_2_ + e^−^ = O_2_^−^). As a result, the YZO-NR will have a plenty of negatively charged oxygen trap sites at its surface (Figure 4a).

When the UV light is illuminated onto the YZO-NR, electron-hole pairs (EHPs) are generated inside the nanorod (Figure 4b). Then, the large concentration of photo-generated holes would be trapped at negatively ionized oxygen molecules, whereas the photo-electrons could contribute to the photocurrent (i.e., 2O_2_^−^ + 2EHP → 2O_2_^−^ + 2h^+^ + 2e^−^ → 2O_2_ + 2e^−^). Consequently, such a trapping behavior will result in the deficiency of photo holes in the whole device system; thus, the photocurrent may seldom increase even under light illumination. However, the latter one (i.e., moderate Y concentration) contains a small concentration of negatively ionized oxygen molecules because of rare V_O_ (Figure 4c). In this case, only the small portion of photo holes would recombine with negatively ionized oxygen molecules; then, the rest of photo holes would remain free (O_2_^−^ + 2EHP → O_2_^−^ + 2h^+^ + 2e^−^ → O_2_ + 2e^−^ + h^+^) (Figure 4d). Those residual photo holes can also contribute to the carrier transport as free holes. Eventually, the photocurrent will therefore considerably increase under light illumination.

**Figure 5:**
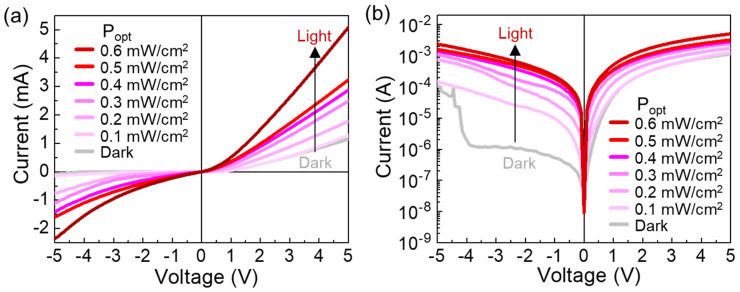
I–V characteristics of the Y3 device under UV illumination with various P_opt_: (**a**) linear scale and (**b**) semi-logarithmic scale.

**Figure 6:**
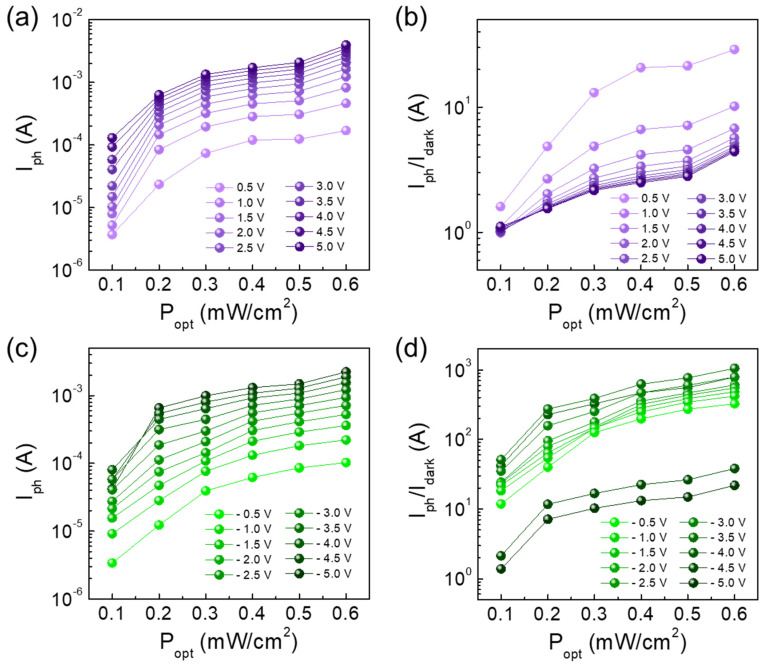
Photoresponse characteristics at various bias voltages of the Y3 device: (**a**) I_ph_ and (**b**) I_ph_/I_dark_ as a function of P_opt_ at forward bias states; and (**c**) I_ph_ and (**d**) I_ph_/I_dark_ at reverse bias states.

Based upon all the above, we chose the Y3 sample for further characterizations. Figure 5 displays the I-V characteristic curves of Y3 under UV illumination with various P_opt_ (0.1–0.6 mW/cm^2^). Here, we note that the UV-A (λ = 365 nm) was used in this experiment because we previously observed that the short penetration depth of the shorter wavelength-UV-light (e.g., UV-B and UV-C) led to the weak photoresponse characteristics in ZnO [58]. As P_opt_ increases from 0.1 to 0.6 mW/cm^2^, the current level gradually increases in both ±V regions (Figure 5a). The increased photocurrent can be ascribed to the excellent UV adsorption characteristics of both PFO and YZO-NR (see Supplementary Materials, Figure S1). Particularly, the device exhibits a drastic increase in its photocurrent at the reverse bias region (Figure 5b). When P_opt_ = 0.6 mW/cm^2^ and V = −3.5 V, the current was increased up to ~1.3 mA from the dark current of ~1 μA. This is an indicative of the high photo-to-dark current ratio (>10^3^) of the Y3 device. To assess the P_opt_ dependence of the steady-state photocurrent (i.e., I_ph_ = I_light_ − I_dark_) and its corresponding steady-state on/off ratio (i.e., I_ph_/I_dark_), we extracted the I_ph_ and I_ph_/I_dark_ values from the P_opt_-dependent I–V curves, and plotted those as a function of P_opt_ (Figure 6). At the forward bias states, I_ph_ and I_ph_/I_dark_ monotonically increases with increasing P_opt_ (Figure 6a,b). However, at the reverse bias states, both I_ph_ and I_ph_/I_dark_ exponentially increase with increasing P_opt_ because of the clear rectification characteristics in the PFO/YZO-NR heterojunction diode (Figure 6c,d). Accordingly, the high magnitude of I_ph_/I_dark_ (>10^3^) is achievable at V = −3.5 V.

**Figure 7:**
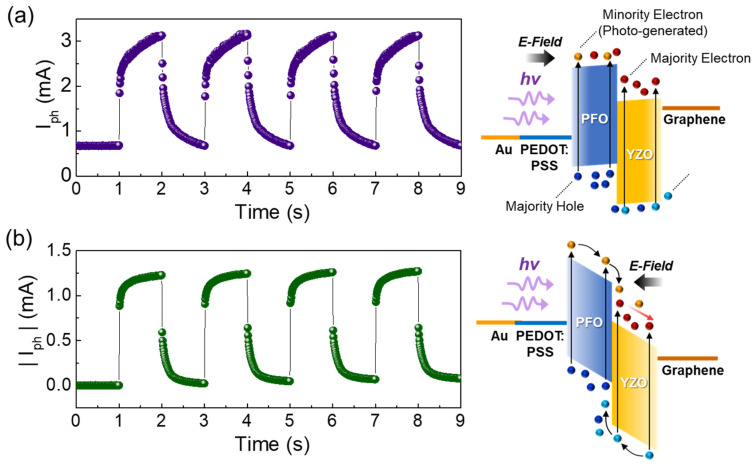
Photoresponse transient waveforms of the Y3 device under UV illumination (P_opt_ = 0.6 mW/cm^2^): (**a**) under forward bias at +3.5 V and (**b**) under reverse bias at −3.5 V. The right-hand-side insets of (**a**) and (**b**) represent the energy band diagrams and their corresponding photocarrier transport behaviors at forward and reverse bias states, respectively.

Next, we examined the photoresponse transient characteristics of the Y3 device at a UV light switching frequency of 0.5 Hz (P_opt_ = 0.6 mW/cm^2^). As shown in Figure 7, the device reveals the typical I_ph_ transient waveforms under both the forward and reverse bias states. However, the on-state current is more stable at reverse bias (Figure 7a) than that at forward bias (Figure 7b). Accordingly, the rising time (τ_r_ = 0.13 s) at reverse bias is faster than that at forward (τ_r_ = 0.51 s). Similarly, the decay time (τ_d_ = 0.22 s) is also shorter at the reverse bias state, compared to that at the forward bias state (τ_d_ = 0.44 s). Such a bias dependence of the photoresponse time is thought as associating with the difference in carrier transport mechanisms under forward and reverse bias conditions, as explained below.

At thermal equilibrium, a large internal electric-field would be created along the direction from YZO-NR to PFO because the work function energy of PFO is greater than that of YZO-NR (see Supplementary Materials, Figure S2). Under the forward bias condition, therefore, the large portion of the applied voltage should be spent to release the large internal electric-field. In this case, the residual potential barrier at the PFO/YZO-NR interface would impede the drift of photocarriers while a diffusion process could easily take place at the PFO/YZO-NR heterojunction (Figure 7a, inset). At the initial stage of light illumination, the injection barriers at the conduction band and the valence band would cause the accumulation of photo-generated electrons and holes at YZO-NR and PFO, respectively. If one keeps on illuminating the UV light, more of photocarriers will be accumulated at the PFO/YZO-NR hetero-interface; then, the accumulated electrons and holes would jump over the injection barriers so as to diffuse into PFO and YZO-NR, respectively. On the other hand, the reverse bias voltage would give rise to the increase in the electric field across the heterojunction, leading to the drift conduction of photocarriers (Figure 7b, inset). According to Ramo’s theorem [59], the photoresponse time relies on the photocarrier velocity; hence, the fast photoresponse could be achievable when the carrier transport is mostly governed by the drift carrier action. Therefore, it can be inferred the faster photoresponse at reverse bias in our Y3 device to arise from the drift conduction of photocarriers.

Finally, we calculated some key parameters of the Y3 device. For PDs, the quantum efficiency (*η_QE_*) is one of the most important figure-of-merits, indicative of an ability to convert the incident photons to the electronic carriers. The *η_QE_* value can be easily determined in terms of I_ph_ and P_opt_ [60] by using the following equation:

(1) $$\eta_{QE}=\frac{I_{\mathrm{ph}}}{P_{\mathrm{opt}}}\times \frac{h\upsilon }{q}\left(\times 100\%\right)$$

where *hν* is the photon energy of the incident light, and *q* is the elementary unit charge. Using Equation (1), we obtained a high magnitude of *η_QE_* ~ 690% at −3.5 V under UV illumination with P_opt_ of 0.6 mW/cm^2^. We ascribe such a high value of *η_QE_* to the decreased photocarrier trap-sites because, as discussed earlier, the incorporation of Y dopants may effectively compensate the oxygen-related surface defects in YZO NRs. According to the literatures [61,62], *η_QE_* is directly relevant to both responsivity (*R*) and detectivity ($D^{*}$) as follows:

(2) $$R=\eta_{QE}\frac{q}{h\upsilon }$$

(3) $$D^{*}=\frac{R}{\sqrt{2qJ_{dark}}}$$

where *J*_dark_ is the current density at dark space. Due to the low value of *J*_dark_ (=1.06 × 10^−6^ A/cm^2^) and the large magnitude of *η_QE_* for our Y3 device, $D^{*}$ was computed to be 3.34 × 10^12^ cm·Hz^1/2^·W^−1^ by using Equations (2) and (3). This value is greater than others [50,51], except for that of the PFO/ZnO PD consisting of the avalanche type of multiple PFO/ZnO stacks [52] (See Table 1). In addition, the high magnitude of *η_QE_* is also closely related to the photocarrier lifetime (τ_PC_) and the photocarrier diffusion length (*L*_PC_) as follows [60,63]:

(4) $$\tau_{\mathrm{PC}}≃\eta_{QE}\cdot \tau_{\mathrm{TR}}$$

(5) $$L_{\mathrm{PC}}=\sqrt{D_{\mathrm{PC}}\cdot \tau_{\mathrm{PC}}}$$

where τ_TR_ and *D*_PC_ are the transit time and the diffusion coefficient of the photocarrier. Using the average values of photocurrent and time delay during four pulses in Figure 7b, *η_QE_*_(ave)_ and τ_TR(ave)_ were calculated to be 660% and 0.17 s, respectively. Therefore, we could estimate τ_PC_ of Y3 at reverse bias to be ~1.16 s. Since the larger value of τ_PC_ is responsible for the longer length of *L*_PC_, one can expect our Y3 device to possess a long *L*_PC_. We accredit the large magnitude of *L*_PC_ to the conformation of the *β*-phase PFO molecules. As mentioned earlier, *β*-phase backbone-like PFO has a flatter geometry of closely-fitted polymer chains. Since *β*-phase PFO has a higher carrier mobility than that of glassy PFO [44], the migration of photocarriers can effectively occur in the organic-inorganic hybrid heterojunction. Consequently, we can conclude that the excellent UV photoresponse characteristics arise from the synergetic effects from both the incorporation of Y (i.e., compensation of surface defects) and the formation of *β*-phase PFO (i.e., increase of carrier conductivity).

## 4. Summary and Conclusions

We fabricated the high-performance PFO/YZO-NR organic-inorganic hybrid heterojunction PD that shows the outstanding UV photoresponse characteristics. The device recorded a high magnitude of *η_QE_* to be ~690% even under low-power UV illumination (P_opt_ = 0.6 mW/cm^2^). Accordingly, a high I_ph_/I_dark_ ratio ~10^3^ and the large $D^{*}$ value ~3.34 × 10^12^ cm·Hz^1/2^·W^−1^ were accomplished at the reverse bias voltage of −3.5 V. In addition, the device revealed stable and fast UV photoresponse characteristics in its photocurrent transient waveforms (i.e., τ_r_ = 0.13 s and τ_d_ = 0.22 s). We interpreted these excellent photoresponse characteristics as resulting from both the increased carrier mobility via forming β-phase PFO and the decreased VO via Y-doping into YZO-NRs. The results pronounce that the β-phase p-PFO/n-YZO-NR organic-inorganic hybrid heterostructure could be a good choice for demonstrating a high-performance UV PD.

## Figures and Tables

**Table 1:** Comparison of the photoresponse characteristics for various PFO/ZnO-based organic-inorganic hybrid heterojunction PDs.

Materials and Structures	λ_UV_ (nm)	P_opt_ (mW/cm^2^)	V_B_ (V)	τ_r_ (s)	τ_d_ (s)	η_QE_ (%)	I_ph_/I_dark_	R (A/W)	D (cm·Hz^1/2^·W^−1^)	Ref.
ZnO NRs/PFO	365	0.6	−3.5	0.13	0.22	690	>10^3^	2.03	3.34 × 10^12^	This work
ZnO NRs/PFO	300		−1			70	~10^2^	0.18	~1 × 10^12^	[50]
ZnO TF/PFO		0.01	2				~10^1^	0.2	>3 × 10^10^	[51]
ZnO NP/PFO	350	1	−10		43 m	2816	>10^3^	4.17	4.93 × 10^12^	[52]

Note: NRs, Nanorods; TF, Thin film; NP, Nanoparticle; λ_UV_, Wavelength of UV light; V_B_, Bias voltage.

## Supplementary Materials

The following are available online at https://www.mdpi.com/2079-4991/10/8/1486/s1, Figure S1: Optical absorption spectra of PFO, YZO-NRs, and PFO/YZO-NRs; Figure S2: Energy band diagrams of the PFO/YZO-NR heterojunction: (a) before contact, (b) at thermal equilibrium, (c) under forward bias, and (d) under reverse bias. E_F_, E_c_, and E_v_ in (a) denote the Fermi level, conduction band, and valence band, respectively.
