# Supplementary material for: Highly Sensitive UV Photodiode Composed of β-Polyfluorene/YZnO Nanorod Organic-Inorganic Hybrid Heterostructure
Source: Nanomaterials (Basel). 2020 Jul 29;10(8):1486. doi: 10.3390/nano10081486 (PMC7466385; doi:10.3390/nano10081486)
Supplement: Supplementary file 1 [file nanomaterials-10-01486-s001.pdf]

nanomaterials

Supplementary Materials

Highly Sensitive UV Photodiode Composed of β-Polyfluorene/YZnO Nanorod Organic-Inorganic Hybrid Heterostructure

Youngmin Lee [1], Soo Youn Kim [2], Deuk Young Kim [1,2] and Sejoon Lee [1,2,*]

[1] Quantum-Functional Semiconductor Research Center, Dongguk University - Seoul, Seoul 04623, Korea
[2] Division of Physics & Semiconductor Science, Dongguk University - Seoul, Seoul 04623, Korea
[*] Correspondence: sejoon@dongguk.edu; Tel: +82-2-2260-3946; Fax: +82-2-2260-3945

Optical Absorption Properties of PFO, YZO-NRs, and PFO/YZO-NRs

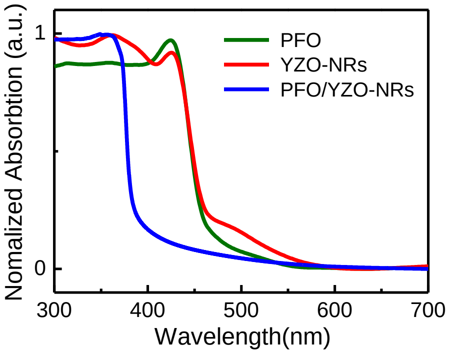

Figure S1. Optical absorption spectra of PFO, YZO-NRs, and PFO/YZO-NRs.

As shown in Figure S1, all of the samples show the high optical absorbance at the wavelength regions below their cut-off wavelengths. Namely, the materials may effectively absorb the photon energies when the light energy is greater than their band-gap energy values.

Energy Band Diagram of PFO/YZO-NR Hybrid Heterojunction

According to the literatures, the work-function energy (Φ) values of Au, PEDOT:PSS, and graphene are 5.1 [1], 5.2 [2], and 4.5 eV [3], respectively; and the electron affinity ($q\chi$) values of PFO and YZO are 3.0 [4] and 4.1 eV (for host material - ZnO) [3], respectively (Figure S2a). In addition, the Φ values of PFO and YZO are known to be ~5.0 [5] and ~4.3 eV (for host material - ZnO) [6]. Based upon Anderson's model [7], at thermal equilibrium (Figure S2b), the heterojunction would be eventually created at the PFO/YZO interface because of the differences in both Φ and $q\chi$ between PFO and YZO. When an appropriate forward bias voltage is applied to the heterojunction, the electrons and the holes would be accumulated at the PFO/YZO hetero-interface because of the electron and the hole barriers near the edges of YZO's E_c and PFO's E_v, respectively (Figure S2c). At this situation, the electron and hole carrier concentrations might be considerably increased if the UV light is illuminated onto the heterojunction (i.e., photocarrier generation). Then, the carriers could transport *via* diffusion because of the large difference in the carrier concentrations (Figure S2c). In the case of reverse bias at dark space, the current level should be extremely small because of the lacks of both electrons and holes in PFO and YZO, respectively. However, the current level would become drastically increased under UV illumination because the drift of photocarriers could take place due to the high electric-field applied at the PFO/YZO heterojunction (Figure S2d).

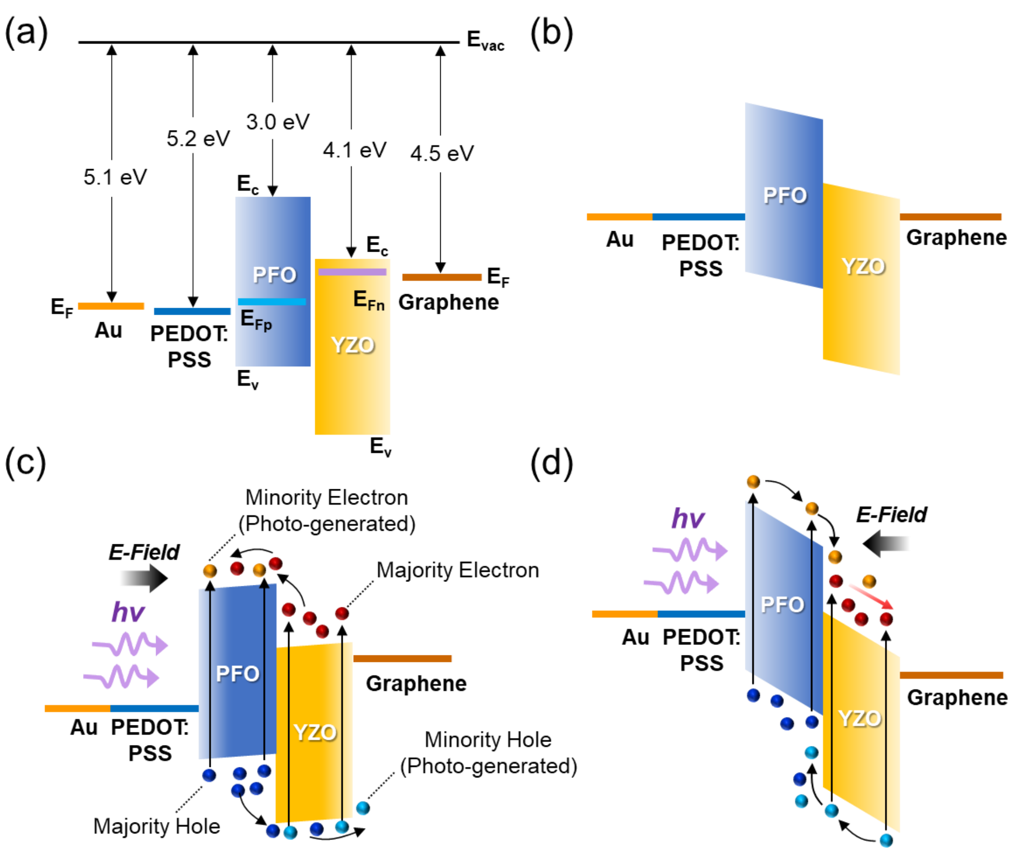

Figure S2. Energy band diagrams of the PFO/YZO-NR heterojunction: (**a**) before contact, (**b**) at thermal equilibrium, (**c**) under forward bias, and (**d**) under reverse bias. E_F, E_c, and E_v in (**a**) denote the Fermi level, conduction band, and valence band, respectively.

References

1. Sze, S.M. *Physics of Semiconductor Devices*, 3rd ed.; Wiley: New York, NY, USA, 2006.
2. Ji, C.H.; Kim, K.T.; Oh, S.Y. Correction: High-detectivity perovskite-based photodetector using a Zr-doped TiO_x cathode interlayer. *RSC Adv.* **2019**, *9*, 40023–40023.
3. Lee, Y.; Kim, D.Y.; Lee, S. Low-Power Graphene/ZnO Schottky UV Photodiodes with Enhanced Lateral Schottky Barrier Homogeneity. *Nanomaterials* **2019**, *9*, 799.
4. Wadeasa, A.; Tzamalis, G.; Sehati, P.; Nur, O.; Fahlman, M.; Willander, M.; Berggren, M.; Crispin, X. Solution Processed ZnO Nanowires/Polyfluorene Heterojunctions for Large Area Lightening. *Chem. Phys. Lett.* **2010**, *490*, 200–204.
5. Rajamanickam, S.A.L.; Mohammad, S.M.; Hassan, Z. Effect of Substrates on Structural, Morphological, Optical and Electrical Characteristics on Poly (9,9-di-n-octylfluorenyl-2,7-diyl) (PFO) Thin Films. *ECS J. Solid State Sci. Technol.* **2020**, *9*, 026002.
6. Gutmann, S.; Conrad, M.; Wolak, M.A.; Beerbom, M.M.; Schlaf, R. Work function measurements on nano-crystalline zinc oxide surfaces. *J. Appl. Phys.* **2012**, *111*, 123710.
7. Milnes, A.G.; Feucht, D.L. *Heterojunctions and Metal.-Semiconductor Junctions*; Academic Press: New York, NY, USA, 1972.

© 2020 by the authors. Submitted for possible open access publication under the terms and conditions of the Creative Commons Attribution (CC BY) license (http://creativecommons.org/licenses/by/4.0/).
